# Route previewing results in altered gaze behaviour, increased self-confidence and improved stepping safety in both young and older adults during adaptive locomotion

**DOI:** 10.1007/s00221-018-5203-9

**Published:** 2018-02-13

**Authors:** Benjamin Thomas Curzon-Jones, Mark Andrew Hollands

**Affiliations:** 1 School of Sport, Exercise and Rehabilitation Sciences, University of Birmingham, Birmingham, B15 2TT UK; 2 Research Institute for Sport and Exercise Sciences, Liverpool John Moores University, Liverpool, L3 5AF UK

**Keywords:** Eye movements, Fear of falling, Gait, Walking, Anxiety

## Abstract

Older adults with falls risk tend to look away prematurely from targets for safe foot placement to view future hazards; behaviour associated with increased anxiety and stepping inaccuracies. We aimed to determine the effectiveness of route previewing in reducing anxiety and optimizing gaze behaviour and stepping performance of younger and older adults. Nine younger and nine older adults completed six walks with three task complexities over two sessions. Each trial used either an isolated stepping target, or a target followed by either one or two obstacles. Participants with eyes closed, on hearing a signal, opened their eyes and initiated walking (go trials) or stood previewing the route for 10 s before starting (preview trials). Kinematic data were collected using a Vicon motion analysis system. Gaze behaviour was recorded using a Dikablis eye tracker. On average, both older and younger adults fixated the target for significantly longer during walking when they had previewed the route than when they had not. Self-confidence scores were also significantly higher following ‘preview trials’ than ‘go trials’. Stepping performance significantly improved following route previewing (reduced Medial lateral foot placement variability for both groups and reduced anterior/posterior foot placement error in older adults only). These findings implicate route previewing as a potential intervention to increase self-confidence and reduce the risk of tripping in older adults.

## Introduction

Visual scanning of the environment to identify obstacles and traversable paths is essential for pedestrians to safely move through our cluttered world. Visual information is continuously gathered and processed to maintain balance and generate the most appropriate locomotor adaptations to ensure safe and efficient travel, e.g., changing direction, stepping over obstacles and placing the feet on stable areas of the terrain. Walking individuals continuously redirect their direction of gaze via rapid eye movements (saccades) to bring environmental features of interest onto the fovea; the area of the retina with the greatest sensitivity. A close timing relationship between saccade onset to fixate a stepping target and swing phase initiation of the targeting limb has been demonstrated in young individuals stepping on illuminated targets (Hollands et al. 1995; Hollands and Marple-Horvat 1996, 2001). This consistent coupling of eye and stepping movements is thought to represent a feedforward control process that relies on visual information describing target location prior to step initiation to pre-programme step trajectory. Once step trajectory is initiated it is consistent during the lead foot swing phase (Lyon and Day 2005) with visually guided fine-tuning when precision stepping is required during the final part of the swing phase (Reynolds and Day 2005). However, older adults, particularly those characterized as having a higher risk of falling, show greater latencies in both onset of gaze refixation towards a new target, and trajectory deviations when adjusting their steps to target translocation during the swing phase (Young and Hollands 2012a). Therefore, it is likely that age-related reduction in the ability to make online stepping adjustments can partially be explained by delays in central processing in addition to any musculoskeletal decline.

As we age, the relative timing of when we look at environmental features we are stepping over, or onto, changes; presumably to allow more time to plan stepping trajectories (Di Fabio et al. 2003a, b; Chapman and Hollands 2007; Zietz and Hollands 2009; Young and Hollands 2012b). For example, Chapman and Hollands (2007) compared the timing of gaze transfer from stepping targets in two groups of older adults deemed to be at a high-risk or a low-risk of falling, and in young adults. They found that high-risk adults transferred gaze away from a target they were stepping towards earlier than the low-risk group and young adults. This early gaze transfer occurred before foot contact with the stepping target and the extent of early gaze transfer correlated with increased mediolateral foot placement variability. These findings are in line with those of Reynolds and Day (2005) who found that visual occlusion of a pre-planned step at swing onset can lead to decreased step accuracy and increased step variability (Reynolds and Day 2005). This decline in stepping performance suggests that visual information can be used in an online manner to fine-tune foot placement during target stepping.

Gage et al. (2003) showed that anxiety induced by manipulating the postural threat posed to participants (i.e., raising the height of the walking surface) led to decreased performance on a secondary task (Gage et al. 2003). They concluded that anxiety led to a greater allocation of attentional resources to the walking task. It has been recently shown that premature transfer of gaze from a current stepping target to fixate future obstacles observed in a group of high-risk older adults was associated with self-reported anxiety, and led to inaccurate steps (Young et al. 2012). Encouragingly, instructing older adults to keep looking at a target until after foot contact during precision stepping improved stepping performance, demonstrating a causal link between early gaze transfer and stepping inaccuracies (Young and Hollands 2010). However, in a fixed laboratory environment, there are no unexpected variables to adapt to, and instructing older adults to fixate their current steps during daily activities might not be a practical method of reducing falls risk when external factors require attention. A better approach would arguably be to address the cause of premature gaze transfer (e.g., anxiety or fear of falling) rather than the symptom.

Young, Wing and Hollands (2012) showed that when facing a target followed by two obstacles, low-risk older adults (who self-reported low anxiety) frequently transferred visual fixation between each of the stepping constraints during their entire approach (Young et al. 2012). However, high-risk older adults (who self-reported higher anxiety) demonstrated a different visual strategy, by fixating the initial target for the majority of their approach toward it, and fixating the subsequent constraints on significantly fewer occasions and for shorter durations compared to older adults without anxiety (Young and Williams 2015). There was also a clear correlation between the number of obstacle fixations and the extent to which older adults transferred their gaze from the target prior to foot contact (i.e., trials in which participants fixated obstacles on fewer occasions, coincided with earlier gaze transfer from the target). These findings suggest that early gaze transfer may be a function of a reduction in the extent that individuals look ahead or preview the upcoming terrain.

We hypothesise that altered gaze behaviour observed in high-risk older adults is due to an anxiety-mediated reduction in the extent to which they preview their walking environment.

This study aimed to assess if: (1) previewing a walking route prior to walking results in changes to older adult gaze behaviour during walking to more closely resemble that of younger adults, working on the assumption that younger adults will have low anxiety and high self-confidence and show optimized visuomotor behaviour, (2) whether changes to gaze behaviour are mediated by state anxiety, and (3) whether any changes to gaze behaviour resulting from previewing are accompanied by improvements in stepping accuracy.

We predicted that route previewing would reduce the frequency and extent of premature gaze transfer from a stepping target in older adults and result in more accurate and less variable stepping. We also predicted that changes to gaze behaviour would be accompanied by a reduction in anxiety and increased self-confidence.

## Methods

### Participants

Nine healthy younger adults and nine community-dwelling healthy older adults were recruited to take part in this study. Young adults were volunteer PhD students from the University of Birmingham’s Sport and Exercise Sciences department (23–29 years old). Older adults (65–87 years old) were recruited from local-assisted living homes, and from poster advertisements placed around the local area. Older adults were compensated £20 for their time plus travel expenses. All participants received a study information sheet prior to attending the lab and signed consent forms on arrival stating that they understood the study, what was required of them, and that they could drop out at any time. Full ethics approval was granted by the University of Birmingham Ethics Committee for the study.

Participants were excluded if they had any self-reported musculoskeletal or neurological impairment, or if they were on prescription medication for anxiety or vestibular problems. The use of corrective lenses was allowed in this study if the participant usually wore them for everyday locomotion, however, participants were excluded if they wore bifocals or varifocals due to incompatibility with the Dikablis head-mounted eye tracker, and their suitability for lower-field walking tasks (Lord and Dayhew 2001).

The following visual and psychophysiological tests were completed prior to any walking trials:

Snellen visual acuity test, Pelli–Robson test for contrast sensitivity, Berg Balance test, Timed up and go task, Falls efficacy scale I, Activities Specific Balance Confidence (ABC) Scale, Trail making test (A&B), Mini-mental state examination, 28-item General Health Questionnaire. The results of these tests together with general participant characteristics are summarised in Table 1.

**Table 1:** General participant characteristics and test scores

Measure: mean (SD)	Young adults (*n* = 9)	Older adults (*n* = 9)
Age (years)	25.44 (1.81)	77 (8.29)
Height (cm)	177.44 (7.30)	162.67 (10.22)
Weight (kg)	75.24 (8.50)	67.60 (9.00)
Body mass index	23.54 (1.45)	25.56 (2.67)
Shoe length (cm)	29.11 (2.42)	27.11 (1.62)
Shoe width (cm)	10.44 (1.16)	9.83 (0.87)
Snellen visual acuity (min score)		
Left eye only	≥ 20/30	≥ 20/50
Right eye only	≥ 20/30	≥ 20/50
Both eyes	≥ 20/20	≥ 20/40
Pelli–Robson contrast sensitivity score (max 2)		
Left eye only	1.73 (0.13)	1.48 (0.19)
Right eye only	1.78 (0.12)	1.5 (0.20)
Both eyes	1.88 (1.11)	1.74 (0.19)
Berg balance (/56)	56 (0)	52.78 (6.51)
TUG test (s)	7.45 (0.36)	11.11 (2.33)
FES-I (/48)	17.33 (1.00)	21.22 (7.07)
ABC (%)	98.19 (2.11)	88.46 (19.10)
Trail making A (s)	21.98 (4.13)	47.66 (24.67)
Trail making B (s)	42.12 (6.10)	148.77 (142.7)
ΔTrail making (s)	20.14 (4.26)	101.29 (112.29)
Mini-mental state (/30)	29.78 (0.44)	27.33 (1.94)
GHQ-28 (/21 each)		
Somatic symptoms	4.44 (2.65)	4.56 (2.92)
Anxiety/insomnia	4.22 (2.86)	5.44 (2.65)
Social dysfunction	4.22 (2.44)	5.78 (0.97)
Severe depression	0.33 (1.00)	0.56 (1.33)

### Data collection

An adapted version of the Vicon lower-body plug-in gait model was used with an additional two markers on the medial and lateral sides of each foot, and the toe markers were moved forward to the upper front edge of each shoe. A 13-camera Vicon MX motion capture system was used to record body kinematics with a sampling frequency of 100 Hz (Oxford Metrics, England).

A head-mounted mobile Ergoneers Dikablis monocular eye tracker was used to record spatial and temporal gaze behaviour, sampling at 25 Hz. The Dikablis system generated a video image of the visual scene with gaze direction superimposed as a crosshair for each trial. Saccadic timings were recorded using a BlueGain EOG Biosignal Amplifier (Cambridge Research Systems, England), sampling at 1000 Hz across separate vertical and a horizontal channel. This signal was synced to the Vicon kinematic recordings via a near infrared input channel using a custom Matlab script (The Mathworks Inc., US). Heart rate was recorded using an Oregon Scientific strapless heart rate monitor (Oregon Scientific, UK).

### Protocol

**Fig. 1:**
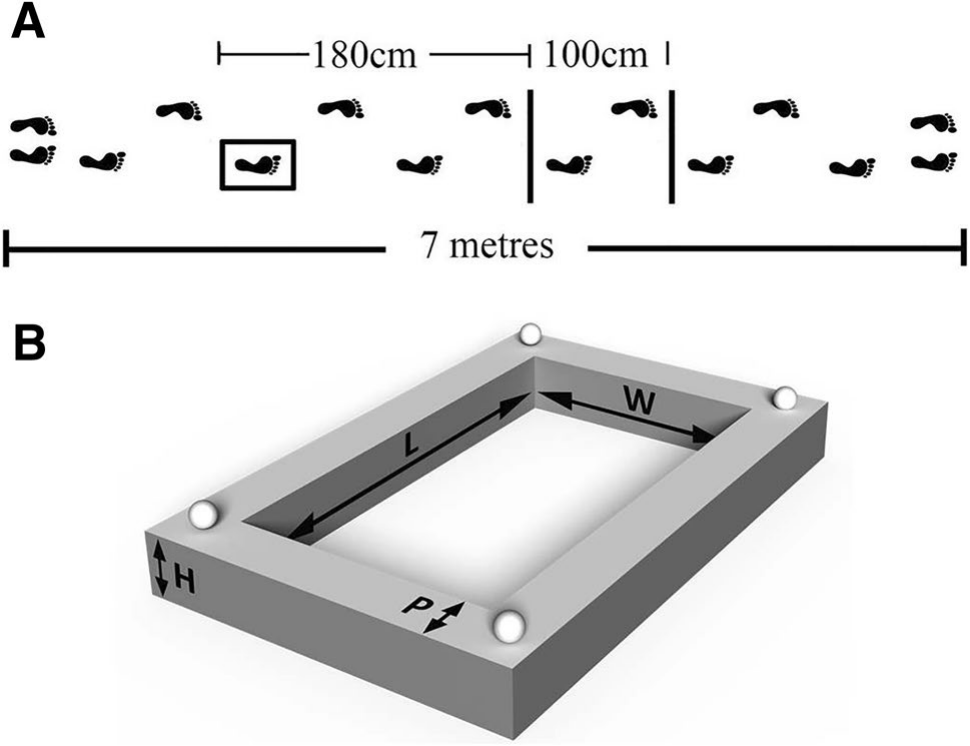
**a** Schematic of the walking task. Participants had to place their right foot into a target box and then step over either none, one or two additional obstacles when present. **b** A 3D representation of the stepping target. Dimensions: *L* = 8 cm + shoe length, *W* = 8 cm + shoe width, *H* = 4 cm, *P* = 5 cm. The box was black and its sides collapsed if stepped on. The four spheres on each of the corners represent reflective kinematic marker positions

Participants were required to walk a 7-m path starting with their right foot. On their second right step, they had to accurately step into a target box and then over a varying number of obstacles until they reached the end of the course (Fig. 1a).

The target box was a raised black rectangular outline that was 4 cm high and 5 cm wide all the way around. The length of the inside stepping area was 8 cm plus the length at the longest part of the participant’s right shoe, and the width was 8 cm plus the width at the widest point of the right shoe. This meant that each participant had the same spatial stepping constraints as each other. The target box (Fig. 1b) was made from solid corner blocks, and joined with collapsible sides to reduce the risk of falling if accidentally stepped on.

Participants were instructed to step into the target whilst ensuring that there was an equal amount of space around their shoe and the inside perimeter of box. This could only be achieved if the centre of the foot was aligned with the centre of the target in both the *M*/*L* and *A*/*P* directions. The obstacles used were 60 cm × 2 cm × 20 cm (width × depth × height) wooden boards with two stabilising blocks at either end to allow it to stand upright. This meant that if the obstacle was knocked in the direction of walking that it would fall flat and not cause a trip or fall. Participants were required to step over these obstacles with their right foot first.

Three task difficulty levels were used: (1) no obstacles following the target box (target only—TO), (2) one obstacle following the target box (one obstacle—OO), and (3) two obstacles following the target box (both obstacles—BO). Participants completed six trials of each difficulty in two separate sessions on the same day, and were allowed four familiarisation trials of each task prior to starting the recorded trials. In each session, participants were required to stand on a start line facing away from the course, then turn 180° to face the course with their eyes shut and, when instructed, either open their eyes and start immediately (‘Go’ trials), or open their eyes and preview the route for 10 s before being told to start walking (‘Preview’ trials). When previewing the route, participants were told to plan their steps and examine the course to step most accurately and avoid the obstacles.

Participants were instructed during familiarisation trials to initiate gait immediately after opening their eyes. The point was stressed that they must open their eyes and start walking immediately. If during familiarisation trials participants were seen to delay gait initiation, the instructions were repeated until they started walking straight after opening their eyes.

Preview and Go trials were completed in separate sessions on the same day, and their order was randomised and counterbalanced across all participants. The three trial difficulty blocks within each session (TO, OO and BO) were also completed in a random order.

Following each set of six trials, participants’ heart rate was recorded and they were asked to complete a State Anxiety Inventory (SAI) of six questions, and the Immediate Anxiety Measurement Scale (IAMS) in relation to how they felt during the trials they had just completed. These responses were later compared against baseline measures taken at the start of the session following the familiarisation trials. Baseline measures were taken prior to each session (go and preview sessions). This means that a baseline measure was taken approximately 1.5 h into testing (session 1 baseline) and again at approximately 2.5 h in to testing (session 2 baseline). We believe that participants were familiar with the environment by the time baseline measures were taken, and that the balanced presentation of the sessions controls for any further effect of lab familiarisation on state anxiety.

### Data analysis

Position data from a video capture frame midway between foot contact and toe off were used to identify stepping accuracy. The centre point of the box was determined by finding the average of the four corners’ (*x, y*) coordinates. The centre of the foot was found similarly but using the (*x, y*) coordinates of the four foot markers. To account for any misalignment of the target box within the Vicon capture field, anteroposterior and mediolateral displacements were calculated relative to the target box orientation. To achieve this, a line crossing through the midpoint of the rear edge and centre of the box was calculated. The *x*-coefficient and *y*-intercept from that line were applied to the foot centre coordinates to create a parallel line running through the centre of the foot. Another perpendicular line running through the centre of the box, and subsequently its point of crossing the central foot line, were calculated. Pythagoras’ theorem was then used to determine the anteroposterior, and mediolateral displacement of the foot relative to the target box. Both the mean (step accuracy) and the standard deviation (step variability) of target box steps were analysed.

Occurrences of the right foot visibly contacting the target box were recorded as frequency per set of six trials.

Foot contact and toe off events within the target box were identified using the heel and toe markers’ vertical acceleration profile.

Trials were labelled in Vicon Nexus using custom models for each study. Each marker’s (*x, y, z*) position coordinates were then exported to a CSV format, and then analysed in Matlab (The Mathworks, Inc. MA, USA).

Data were filtered with a zero-phase fourth-order Butterworth Filter with a cut-off frequency of 7 Hz. An adapted method of foot contact identification used by O’Connor et al. (2007) was used to detect separate heel and toe contact rather than general foot contact. Foot contact and toe off events were identified using the vertical acceleration profile of heel and toe markers (O’Connor et al. 2007). A large velocity acceleration peak in the respective traces coincided with heel and toe contact with the floor. To isolate the foot contact peak, a window of 400 ms following the heel crossing the rear edge of the target box was used to identify the range of data in this area for both heel and toe markers. A window size of 400 ms was chosen as it would include the contact peaks, but would not include the peaks generated by toe off and heel off events. 30% of the *y*-axis range of this 400 ms window was chosen to be a suitable cut-off point to isolate the contact peaks. If contact peaks were not identified, or if multiple peaks occurred in the isolated section, the trials were flagged for manual data extraction. The local maximum of the earliest occurring peak identified the foot contact time and also the participants stepping strategy. It was noted that some participants stepped into the target box with their toe first instead of heel first; the frequency of this behaviour was recorded.

Heel off and toe off events were identified as the second peaks that exceeded 30% of the *y*-axis range within the 400 ms window following the heel crossing the rear edge; however, only the toe off peak was necessary as it would be impossible for the toe to leave the ground before the heel during normal gait. This method allowed differentiation of the target box stepping strategy used by each participant on a trial-to-trial basis. Participants either made floor contact with their heel or toe first. The percentage of toe-first steps was recorded for each set of six trials.

Stance duration inside the target box was also calculated as the time between foot contact and toe off.

A 3 × 2 × 2 (task difficulty × preview condition × age group) mixed design ANOVA was used to identify any main effects or interactions of stepping characteristics relating to the target box. Leading and trailing foot toe clearance on the near obstacle was measured for trials where the near obstacle was present.

Spatial and temporal visual behaviour analysis was carried out using the D-Lab Eye-Tracking suite (Ergoneers GmbH, Germany). Blink artefacts were removed prior to analysis using the software’s in-built algorithm. Three areas of interest (target box, near obstacle and far obstacle) were marked out on-screen in relation to real-world adjacent visual markers identified by the software, and fixation periods within these areas were calculated. Fixation was classified at three frames of video, which is equivalent to 120 ms and falls within the normally accepted range of fixation period (Patla and Vickers 1997). Preview and walking sections were separated into different outputs. Two dependent variables were extracted from these data: (1) total duration spent fixating an area, and (2) percentage of the trial or section spent fixating an area. Mixed design repeated measures ANOVAs were used to analyse the Dikablis eye-tracking data. Within-subject differences of task difficulty were removed from ANOVA analysis as each difficulty had a different number of visual targets to fixate, and comparisons between these would be invalid. Therefore, independent *t* tests were used to analyse between-subject differences in pagereview fixation periods on the target box, near obstacle and far obstacles. The Bonferroni correction was applied to all *p* values analysed in the *t* tests.

Saccadic timings were calculated by temporally synchronising the EOG signal to the Vicon data, then using the previously identified foot contact time as a reference for the appropriate saccadic eye movement, averaged for each set of six trials, and analysed in a 3 × 2 × 2 repeated measures ANOVA.

The anxiety score from the SAI was scored out of a possible 12 points. The Immediate Anxiety Measurement Scale (IAMS) was also used. Any changes in anxiety in the current study would be due to indirect influences; therefore, it was included to examine self-reported anxiety and self-confidence in greater detail. IAMS scores were split in to two sections. Section A was on a Likert scale of 1–7 and related to cognitive anxiety, somatic anxiety and self-confidence. Section B described on a scale of − 3 to + 3 whether participants found their relative presence, or lack of each item, in section A to be debilitative or facilitative. These scores were also based on change from baseline levels and gave six variables for each of the six sets of trials. Change from baseline for heart rate data following each set of trials was also calculated. SAI, IAMS and heart rate data were all analysed using a 3 × 2 × 2 repeated measures ANOVA.

Correlation analysis comparing at least one non-parametric variable (IAMS, SAI, target hit frequency and toe-first stepping percentage) was carried out using Spearman’s Rank correlation. If both variables were parametric (stepping error and variability, stance duration, gaze transfer time and target fixation time) then Pearson’s product–moment correlation coefficient was used. All correlation analyses were two-tailed. *p* values were adjusted using the Bonferroni correction for multiple comparisons; only correlations with a *p* value less than 0.003 are reported.

To control for the potential confounding influence of group-related differences in walking speed, walking speed was added as a covariate for analysis of any dependent variable which significantly correlated with walking speed.

## Results

A summary of all results can be found in Table 2. All values presented in this section are mean ± standard error unless otherwise stated.

**Table 2:** Means and standard errors for anxiety measures, gaze behaviour and stepping performance

Mean ± SD	Go		Preview	
	Younger adults	Older adults	Younger adults	Older adults
Anxiety measures				
IAMS cognitive anxiety	− 0.22 ± 0.42	0.37 ± 1.24	− 0.33 ± 0.48	0.11 ± 0.93
Direction	0.15 ± 0.46	− 0.37 ± 1.52^d^	0.04 ± 0.52	0.26 ± 1.26
IAMS somatic anxiety	− 0.19 ± 0.40	− 0.11 ± 0.58	− 0.26 ± 0.45	0.00 ± 0.96
Direction	− 0.04 ± 0.44	− 0.04 ± 0.98	0.00 ± 0.39	0.07 ± 1.52
IAMS self-confidence^a^	0.30 ± 0.72	− 0.04 ± 1.65	0.37 ± 0.63	0.52 ± 1.55
Direction	0.07 ± 0.27	0.11 ± 1.09	0.07 ± 0.27	0.26 ± 1.35
SAI	0.04 ± 1.45	− 0.04 ± 1.74	− 0.15 ± 1.06	− 0.41 ± 2.52
Gaze behaviour				
Saccade timing (ms)^b^	50 ± 138	− 77 ± 114	44 ± 142	− 86 ± 174
Saccade variability (ms)^a,b^	89 ± 51	151 ± 109	96 ± 78	166 ± 152
Walking target fixation (%)^a^	23.98 ± 6.00	21.71 ± 4.74	26.02 ± 5.92	26.02 ± 4.20
Stepping performance				
*A/P* stepping error (mm)	− 6.66 ± 6.74^c^	− 27.76 ± 12.64^d^	− 7.38 ± 8.22	− 22.64 ± 13.58
*A/P* stepping variability (mm)	14.71 ± 6.48	14.43 ± 5.77	6.09 ± 5.66	7.31 ± 5.89
*M/L* stepping error (mm)	− 7.83 ± 8.06	− 7.40 ± 10.87	− 8.99 ± 6.59	− 11.36 ± 7.92
*M/L* stepping variability (mm)	9.32 ± 2.80	12.03 ± 4.47	3.52 ± 3.94	4.28 ± 4.84
Stance duration (s)	0.77 ± 0.07	0.87 ± 0.11	0.82 ± 0.08	0.96 ± 0.22
Toe-first stepping (% of trials)^b^	24.07 ± 35.00	68.52 ± 38.49	24.07 ± 36.49	70.99 ± 34.77
Leading foot toe clearance (mm)	18.77 ± 4.98	12.49 ± 2.17	17.67 ± 5.83	13.17 ± 4.21
Trailing foot toe clearance (mm)	17.95 ± 6.40	10.92 ± 4.24	15.35 ± 5.29	9.60 ± 7.05
Target hit frequency (per six trials)	0.22 ± 0.51	1.07 ± 1.17	0.04 ± 0.19	0.63 ± 0.97
Walk time	7.47 ± 0.89	9.58 ± 2.14	7.77 ± 0.82	10.33 ± 2.80

N.B. Raw values only—changes to the due to covariance analysis are not presented

^a^sig. overall difference between ‘Go’ and ‘Preview’ trials

^b^sig. overall difference between age groups

^c^sig. difference between age groups within the same session

^d^sig. difference between sessions within the same age group

### Anxiety and self-confidence

**Fig. 2:**
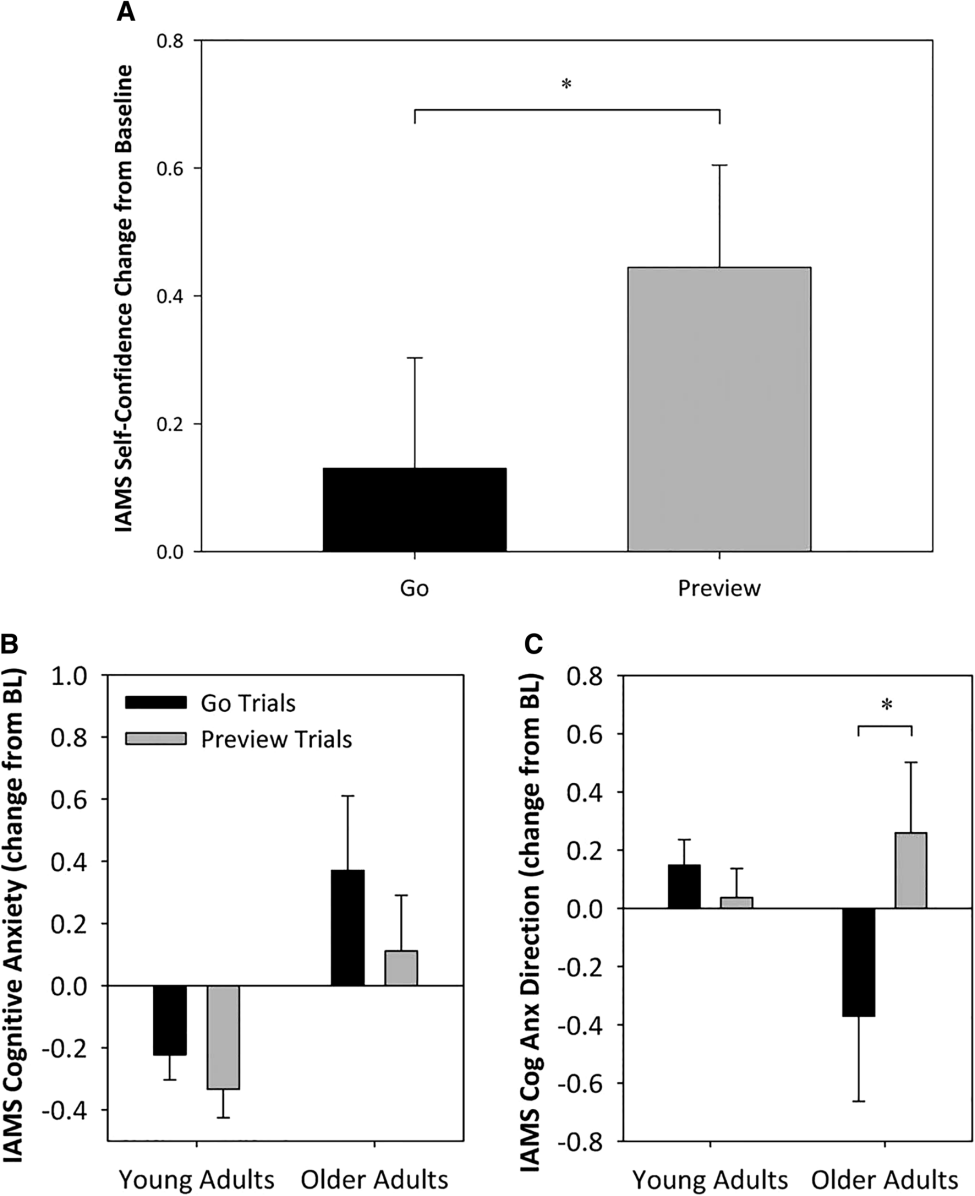
**a** IAMS self-confidence scores as a change from baseline for each session and both age groups. Error bars represent standard errors (SE). *Sig. session difference *p* < 0.05. **b** IAMS cognitive anxiety change from baseline for age and session. **c** The change from baseline measures of the psychological direction that participants perceived their cognitive anxiety to be assisting them with their stepping performance. If it was facilitating performance the score was positive, and if it was debilitating performance the score was negative. Graph (**a**) has been included to show the levels of anxiety to which graph (**b**) was scored. *Main effect of session within the age group, *p* < 0.05. Error bars show standard error

There was a main effect of session on self-reported IAMS self-confidence change from baseline score (*F*_(1,16)_ = 6.84, *p* < 0.05, *ƞ*^2^_partial_ = 0.27). Self-confidence was significantly higher in ‘preview trials’ compared to ‘go trials’ [0.44 ± 0.27 and 0.13 ± 0.30, respectively (mean ± standard error), Fig. 2a].

There were no main effects for somatic and cognitive anxiety IAMS scores (Fig. 2b), however, there was a significant age group × session interaction for cognitive anxiety direction change from baseline (*F*_(1,16)_ = 4.75, *p* < 0.05, *ƞ*^2^_partial_ = 0.23). Older adults rated their current level of cognitive anxiety (regardless of value) to be more beneficial to their stepping performance during ‘preview trials’ than during ‘go trials’ (0.26 ± 0.32 and − 0.37 ± 0.31, respectively), younger adults showed no difference between sessions (Fig. 2c).

There was a main effect of task difficulty on heart rate change from baseline (*F*_(1,16)_ = 3.78, *p* < 0.05, *ƞ*^2^_partial_ = 0.19). Post hoc tests showed that heart rate during TO trials (0.06 ± 1.11 bpm) was significantly lower than the OO (2.1 ± 1.2 bpm) difficulty, but not from BO (1.6 ± 1.4 bpm).

There were no between-subject or within-subject significant differences in scores on the state anxiety inventory.

### Walking characteristics

#### Walking speed

There were main effects of age group, session and difficulty on mean walking speed (*F*_(1,16)_ = 3.78, *p* < 0.05, *ƞ*^2^_partial_ = 0.62, *F*_(1,16)_ = 5.74, *p* < 0.05, *ƞ*^2^_partial_ = 0.26, and *F*_(2,32)_ = 125.62, *p* < 0.001, *ƞ*^2^_partial_ = 0.89). Younger adults were significantly quicker than older adults (0.93 ± 0.04 and 0.74 ± 0.04 ms^−1^, respectively), ‘preview trials’ were slower than ‘go trials’ (0.82 ± 0.03 and 0.86 ± 0.03 ms^−1^, respectively), and each task difficulty was significantly different from the other two, with decreasing speeds as difficulty increase (TO: 0.90 ± 0.03 ms^−1^, OO: 0.84 ± 0.03 ms^−1^, BO: 0.77 ± 0.03 ms^−1^, *p* < 0.001). Walking speed was added as a covariate to the indicated analyses below, to account for any changes in speed between age groups or conditions.

#### Mediolateral foot placement

**Fig. 3:**
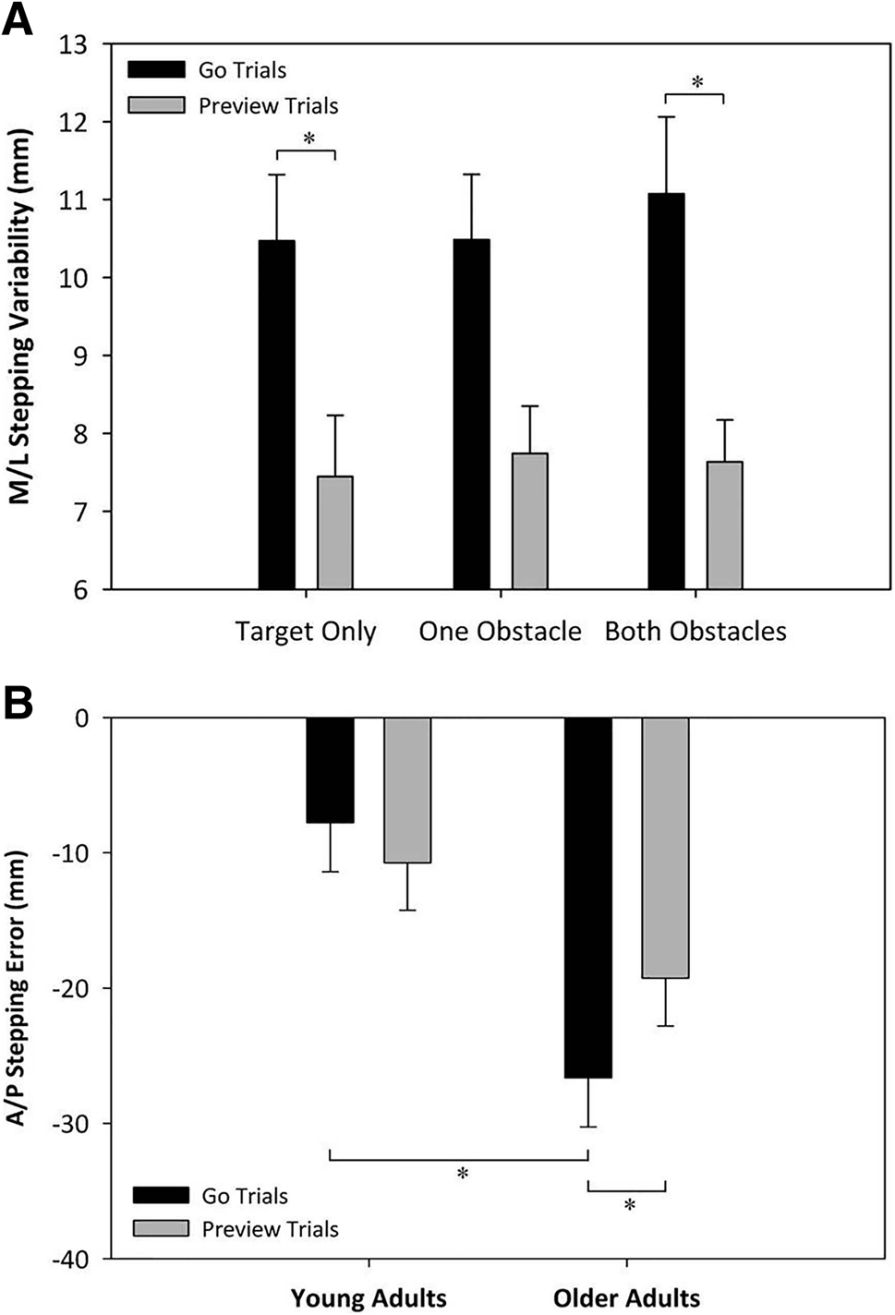
**a** Mediolateral stepping variability of each session within each task difficulty. *Sig. difference between sessions, *p* < 0.05. Error bars represent standard error (SE). **b** Anteroposterior stepping error in each session for younger and older adults. Negative numbers indicate posterior stepping. *p < 0.05 for indicated conditions and groups. Error bars represent standard error

Repeated measures ANCOVA showed a interaction of session and difficulty on mediolateral (*M*/*L*) stepping variability within the target box (*F*_(2,30)_ = 4.12, *p* < 0.05, *ƞ*^2^_partial_ = 0.22). *M*/*L* foot placement variability in the target during ‘preview trials’ was significantly reduced compared to foot placement variability during ‘go trials’ but only in the target only and two obstacle conditions (Fig. 3a).

There were no significant differences between age groups, sessions or difficulties in mean *M*/*L* stepping error.

#### Anteroposterior foot placement

There was a main effect of age group on anteroposterior (*A*/*P*) stepping error (*F*_(1,15)_ = 7.08, *p* < 0.05, *ƞ*^2^_partial_ = 0.47). Older adults (− 23.0 ± 3.3 mm) stepped significantly further back from the target centre than younger adults (− 9.3 ± 3.3 mm). There was also an interaction effect of age and session on *A*/*P* stepping error (*F*_(1,15)_ = 5.30, *p* < 0.05, *ƞ*^2^_partial_ = 0.26). Post hoc tests revealed a significant difference between younger and older adults during go trials, and older adults stepped with significantly less error in ‘preview trials’ compared to ‘go trials’ (Fig. 3b). There was no significant effect of any variable on *A/P* stepping variability.

#### Target box contact frequency

When walking speed was added as a covariate, we found no significant differences between age group, session or task difficulty on target box hit frequency (Table 2).

#### First obstacle toe clearance

An independent t test showed older adults to be significantly shorter than younger adults (*t*_(16)_ = 3.53, *p* < 0.005, *d* = 1.66), therefore, a repeated measures ANCOVA with height and walking speed as covariates was used to compare differences in obstacle toe clearance in OO and BO task difficulties. There was a main effect of difficulty on lead toe clearance (*F*_(1,14)_ = 5.49, *p* < 0.05, *ƞ*^2^_partial_ = 0.28) showed slightly greater toe clearance during OO trials (15.6 ± 0.9 mm) than BO trials (15.4 ± 0.8 mm) when adjusted for height and walking speed. There were no significant differences for age group, session or difficulty on trailing toe clearance with height and walking speed as covariates.

#### Target box step technique

Some participants approached the precision stepping task using a ‘toe-first’ strategy rather than the usual ‘heel-first’ strategy generally observed in normal locomotion (heel contact).

Heel contact always occurred following toe contact, which was evident in the acceleration and vertical position traces and necessary for identification of gait events. In some trials where the rear edge of the target was struck with the heel in a toe-first step, the heel vertical acceleration trace showed lots of noise; these trials were excluded from analysis.

**Fig. 4:**
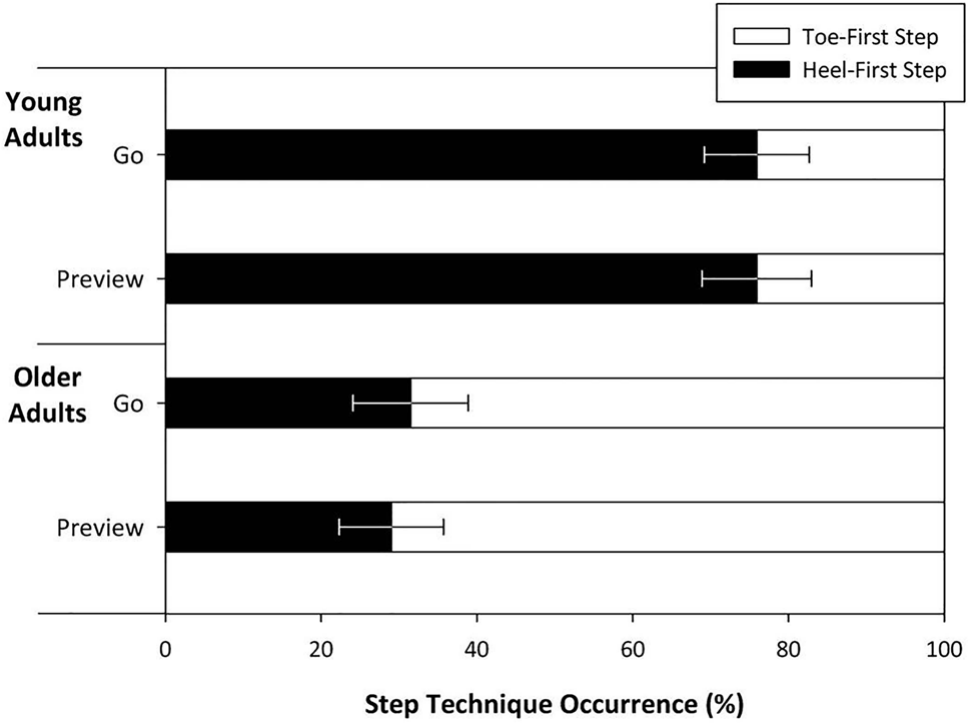
The occurrence of heel-first and toe-first foot contact in the target box as a percentage of each session. There was a significant difference of age, but no significant within-subject variations. Error bars represent standard error

Converting the frequency of ‘toe-first’ steps in each set of trials to a percentage, a repeated measures ANCOVA revealed a main effect of age group on the technique used (*F*_(1,15)_ = 4.79, *p* < 0.05, *ƞ*^2^_partial_ = 0.32). Older adults used the ‘toe-first’ technique in 69.4 ± 13.1% of trials, whereas younger adults only used this approach in 24.4 ± 13.1% of trials. However, previewing the route did not change the step technique used in younger or older adults (Fig. 4).

### Gaze behaviour

#### Target box and obstacle fixations while previewing

Task difficulty was excluded from gaze fixation analysis due to the nature of the task. Previewing trials with more obstacles require participants to fixate more visual targets; therefore, comparisons between these task difficulties would be invalid.

**Fig. 5:**
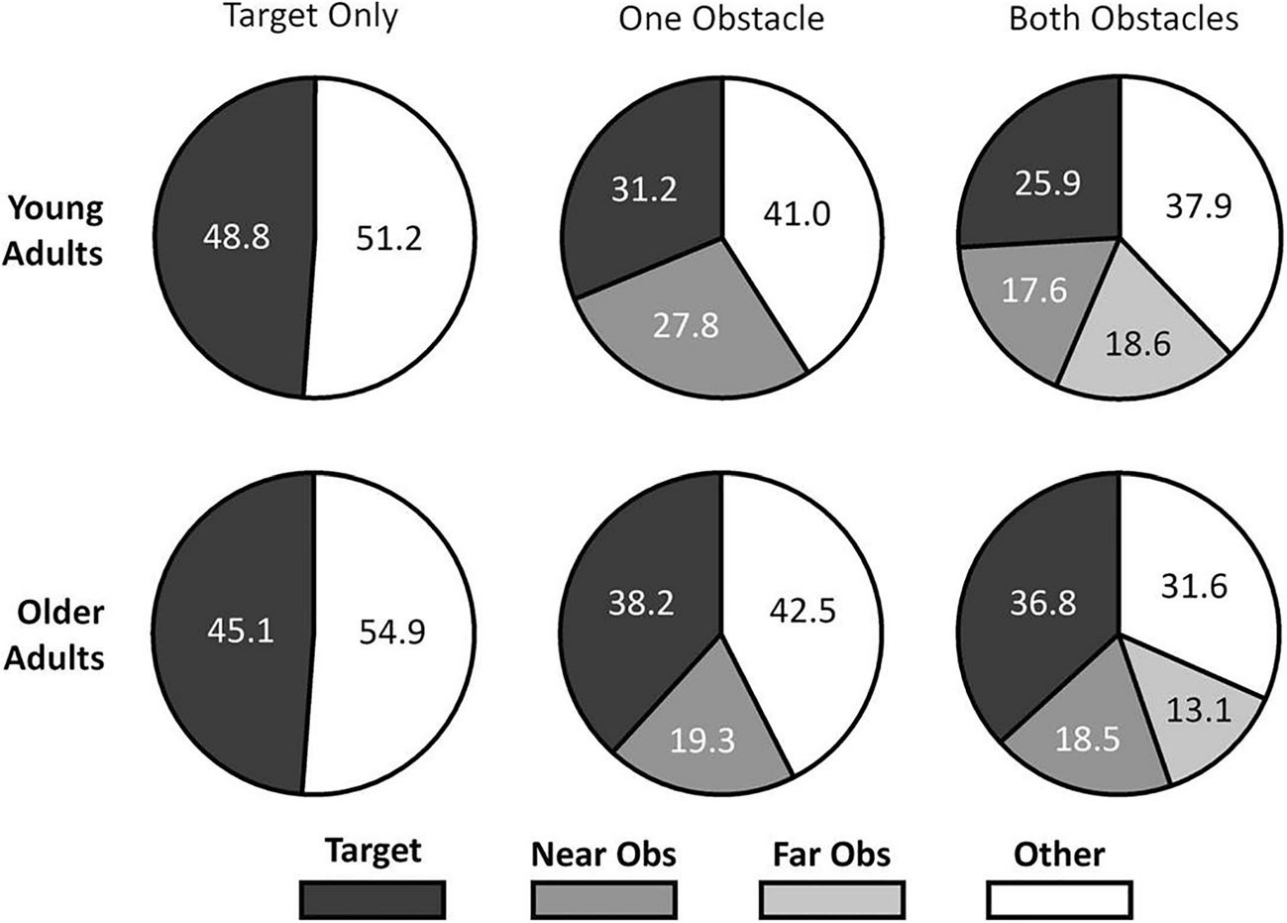
Pie charts summarising the percentage of preview time spent fixating the target, near and far obstacles (where present). Values represent percentage of preview time

There were slight variations in preview times due to the reaction times of verbally signalling participants to start walking, however, these variations were not significantly different between age groups (*t*_(52)_ = 0.50, *p* = .62, *d* = 0.14). Independent *t* tests using the Bonferroni correction for multiple comparisons showed no significant differences of age on target box, near obstacle, or far obstacle fixation times. Figure 5 shows fixation data for both younger and older adults.

#### Target box fixations while walking

**Fig. 6:**
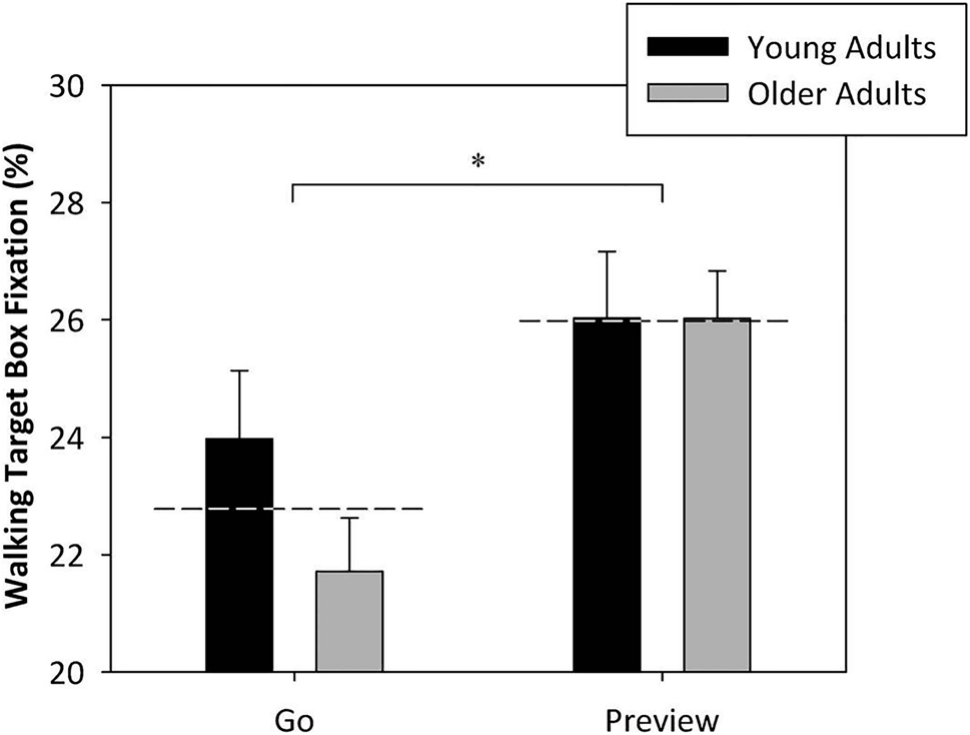
Total target box fixation period while walking and as a percentage of total walk time for younger and older adults in both sessions. Dashed lines represent collapsed mean for each session. *Sig. difference between sessions, *p* < 0.05. Error bars represent standard error

To compare the relative fixation times due to the difference between younger and older adults’ walk times, total target fixation was calculated as a percentage of total walk time. There was a main effect of session on target fixation percentage (*F*_(1,16)_ = 11.67, *p* < 0.01, *ƞ*^2^_partial_ = 0.42), with ‘preview trials’ resulting in a longer fixation percentage than ‘go trials’ (26.0 ± 1.0 and 22.8 ± 1.1%, respectively). There were no significant main effects of age group on the duration of target box fixation expressed as a percentage of total walk time (Fig. 6).

There was also a main effect of difficulty on target box fixation time (*F*_(2,32)_ = 44.65, *p* < 0.001, *ƞ*^2^_partial_ = 0.74). Post hoc analysis revealed that the target box was fixated for longer in the TO (27.7 ± 1.0%) trials compared to OO (23.6 ± 1.1%) and BO trials (22.0 ± 0.8%) (*p* < 0.001).

#### Obstacle fixations while walking

As expected there was a main effect for difficulty on near obstacle total fixation time between OO (12.0 ± 0.8%) and BO (8.9 ± 0.7%) conditions (*F*_(1,16)_ = 28.04, *p* < 0.001, *ƞ*^2^_partial_ = 0.64). TO trials were not included as there was no near obstacle present to fixate on.

There were no significant differences between far obstacle fixations for age group, session or difficulty.

#### Time of gaze transfer from stepping target

There was a main effect of age group on gaze transfer time with respect to foot contact in the target with walking speed as a covariate (*F*_(1,15)_ = 7.44, *p* < 0.05, *ƞ*^2^_partial_ = 0.50). Older adults (− 105 ± 41 ms) transferred gaze significantly earlier than younger adults (71 ± 41 ms).

Standard deviation of gaze transfer time was used as a measure of gaze transfer variability. When walking speed was added as a covariate, there was a main effect for age (*F*_(1,15)_ = 6.44, *p* < 0.05, *ƞ*^2^_partial_ = 0.43). Older adults had a higher gaze transfer standard deviation (167 ± 21 ms) compared to younger adults (84 ± 20 ms) meaning that older adults had a more variable saccade time relative to foot contact. There was also a main effect of session on gaze transfer variability with walk speed as a covariate (*F*_(1,15)_ = 10.88, *p* < 0.01, *ƞ*^2^_partial_ = 0.42). Interestingly, participants showed greater gaze transfer variability during ‘preview trials’ (131 ± 16 ms) than during ‘go trials’ (120 ± 11 ms).

## Discussion

This is the first study to have investigated the effects of previewing a route prior to walking on anxiety and self-confidence levels, stepping performance, and gaze behaviour in younger and older adults performing an adaptive locomotor task. Our primary aim was to identify if a previewing intervention would result in older adults’ gaze behaviour during walking to more closely resemble that of younger adults due to reduced anxiety and result in improved stepping performance. Changes to these variables following route previewing would suggest that the effects of state anxiety on visually guided walking is mediated by reduced visuomotor planning due to inadequate visual scanning of the environment during walking.

### Stepping performance

Route previewing resulted in a significant reduction of *M/L* stepping variability in both younger and older adults and a reduction in *A/P* stepping error in older adults (Fig. 3b). This provides evidence that allowing more time to gather spatial information about the task results in improved stepping performance. We suggest that this is due to improved spatial awareness about the target and obstacles, which allows greater focus on the current stepping tasks.

There was a difference between age groups in percentage of toe-first stepping trials. We found that younger adults used this strategy significantly less often than older adults. We suggest that it is used as a method of trying to judge central stepping from the distance between the inside front of the target box and the front of the stepping foot. However, correlations between toe-first stepping prevalence and anteroposterior stepping error in young adults suggest that this technique might lead to more posterior stepping. If this study was repeated with a target that did not impose any postural threat, such as a box marked on the floor with tape, or a singular mark on the floor, we would not expect to see such a high adoption rate of this toe-first stepping technique. We propose that older adults exercise an increased caution when stepping over the rear edge and into the target area, as a potential trip or fall may be more challenging to recover from compared to their younger counterparts. Guiding a foot to the floor with the toe does not initially commit as much pressure to the step compared with a normal heel strike (Dufek and Bates 1991), and allows for better visual guidance, and easier withdrawal of the foot should an unexpected perturbation occur underfoot. However, the benefits of adopting this toe-first stepping technique appear to be limited, if not detrimental to stepping accuracy, and future research should examine the mechanisms and potential benefits of this selection process in further detail.

### Effects of route previewing on anxiety

Surprisingly, there were no significant differences in IAMS somatic or cognitive anxiety, or state anxiety inventory scores between sessions or task difficulties. We suggest that the absence of a significant change in anxiety, compared to the measured increased in self-confidence is due to the possible variance available for each measure at baseline. If a participant reported low anxiety during the ‘go trials’, the amount that anxiety scores can reduce during ‘preview trials’ is limited. The same could be said for self-confidence, however, due to the novelty of the task, most participants did not report maximum self-confidence during the ‘go trials’. Furthermore, both the younger and older adults in this study would classify as being at a low risk of falling (Podsiadlo and Richardson 1991; Berg et al. 1992), and therefore, would not exhibit as much anxiety regarding this task as previously found in high-risk older adults (Young et al. 2012).

Another possibility is that since the perceived threats to participants balance (obstacles, target, etc.) remain regardless of whether the individual does preview these, then anxiety relating to these threats is unaffected.

In contrast to our assessment of anxiety, we did find a significant increase in the IAMS self-confidence change from baseline score across all participants (Fig. 2a) following route previewing. This indicates that during preview trials, participants were more confident about the walking task, presumably because they had more time to process the goals and constraints of the task and plan accordingly. Zettel et al. (2007) have previously shown that, during unexpected perturbations, previously acquired spatial information about environmental features can be used to guide appropriate motor corrections to maintain balance, e.g., grasping a handrail that was previously fixated (Zettel et al. 2007). Our results suggest that this spatial mapping can occur during the 10 s preview period and results in increased self-confidence and improved task performance. We also showed that older adults perceived their current level of cognitive anxiety to be more beneficial to their stepping performance (Fig. 2c); a trait that has been previously shown to be beneficial to putting performance in golfers (Chamberlain and Hale 2007).

### Previewing gaze behaviour

There were no main effects of age on target or obstacle fixation times during route previewing. However, when interpreting mean total percentage of fixation time (Fig. 5) we can see a trend that older adults fixated with a bias towards more immediate stepping constraints (a greater percentage of time fixating the target box) when compared to younger adults. This trend has previously been identified in high-risk older adults with increased state anxiety compared to low-risk individuals (Young and Hollands 2010, 2012b) and supports the idea that there is an age-related prioritisation of more immediate stepping constraints, even prior to initiating locomotion.

### Walking gaze behaviour

There was an increase in target box fixation as a percentage of total walk time following route previewing (Fig. 6) for both groups. It is encouraging to note that the average fixation time was equivalent for younger and older adults following route previewing, suggesting that the aim of reducing differences in gaze behaviour between groups was at least partially realised. This suggests that during previewing participants were able to gather and store spatial information about the course, and consequently, allow a longer fixation time on more immediate constraints (Zettel et al. 2007). It has previously been shown that balance and locomotion are more attentionally demanding for older adults than for younger adults (Brown et al. 1999; Li et al. 2012). It is possible that previewing the route alleviates some of the older adults’ cognitive load during walking, resulting in gaze behaviour that more closely resembles that of younger adults.

We also found effects of task difficulty on target box and near obstacle fixation time. Target box fixation duration was significantly reduced in OO and BO trials compared to TO presumably because there are more constraints to look at in the more complex tasks. This trend was also observed in near obstacle fixation time, as participants fixated the near obstacle more during OO than BO trials. These results demonstrate that increasing the number of stepping constraints splits the attentional load as would be expected.

We found a main effect of age on gaze transfer time relative to foot contact which was independent of walking speed; older adults transferred gaze significantly earlier than younger adults. This finding is supported by existing literature and suggests that older adults prioritise gathering information about future stepping constraints over visually guiding ongoing stepping actions (Chapman and Hollands 2006, 2007). We also found that older adults exhibited a higher variability in the timing of gaze transfer from the target box compared to younger adults.

Surprisingly, we did not find a significant effect of route previewing on the timing of gaze transfer with respect to foot contact, i.e., older adults still looked away earlier than younger adults during the targeting step even though the total fixation time during the approach was increased. Premature gaze transfer from a stepping target has been causally linked to increased foot placement inaccuracy and variability, and is believed to be driven by anxiety/fear of falling (Young and Hollands 2012b). Although previous studies have generally concentrated their analysis on the timing of gaze transfer, one study has shown similar age- and risk-related effects of total fixation time on the stepping targets as on early gaze transfer (Chapman and Hollands 2007). It is also interesting to note that in the current study route previewing, which resulted in longer target fixations during walking, also had an effect on the variability of the timing of gaze transfer. This finding would suggest, unsurprisingly, that timing of gaze transfer is not independent of total fixation duration, and therefore it is likely that the benefits of looking at targets for longer and until later in the action sequence share a common mechanism. The finding that improvements in stepping performance were achieved even though older adults still looked away prematurely from the target suggests that it was the feedforward planning of the movement that benefited from the increased fixation time rather than online processing to fine-tune the stepping trajectory using foveal vision of the target and/or peripheral vision of the lower limbs. Even though the precise mechanisms through which extended viewing of a target aids performance remain unclear it seems that route previewing prior to walking implicitly promotes this gaze behaviour.

### Limitations and future studies

An obvious limitation in the current study is that we did not study older adults with high levels of anxiety or fear of falling, and therefore are unable to provide evidence that such individuals, who arguably need the intervention most, would benefit from route previewing. Indeed, previous studies have suggested that older adults with fear of falling have a gaze bias to potential threats, and therefore may not comply with the instructions (Staab 2014). However, one could also argue that the best way of addressing the global problem of older adult falls is to create interventions that prevent higher functioning older adults from falling in the first place. Once an injurious fall has occurred then older adults often become anxious and develop fear of falling which can lead to behavioural changes such as maladaptive premature gaze transfer that can, paradoxically, put them at greater risk of subsequent falls (Young and Hollands 2012b). The finding that simple route previewing prior to walking can improve stepping performance in both younger and high-functioning older adults suggests that this may be a viable method of reducing the risks of trips and falls.

Moving forward, there is a clear need for further experimental studies with frail older adults and feasibility studies to gauge the compliance and sustainability of any intervention in older adults and patient groups before any conclusions can be drawn regarding the potential of a route previewing instructional intervention for reducing falls risk in frail individuals in the community.

The finding that a route previewing intervention was effective in improving stepping performance in the groups studied is encouraging. However, the current results also highlight that more work is needed to understand the mechanisms underlying these effects; in particular, the relationships between anxiety, early gaze transfer and fixation duration.

### Conclusions

We have provided evidence that asking adults to preview a walking route prior to gait initiation promotes altered gaze behaviour during walking which results in increased self-confidence and improved stepping accuracy and consistency. Although the mechanisms through which gaze behaviour influences stepping performance remains unclear, it seems likely that route previewing improves the spatial mapping of relevant environmental features that is used to guide locomotor adaptations using feedforward control processes. Irrespective of the mechanism of effect, these findings implicate route previewing as a potential intervention to increase self-confidence and reduce risk of tripping and falling in older adults.
